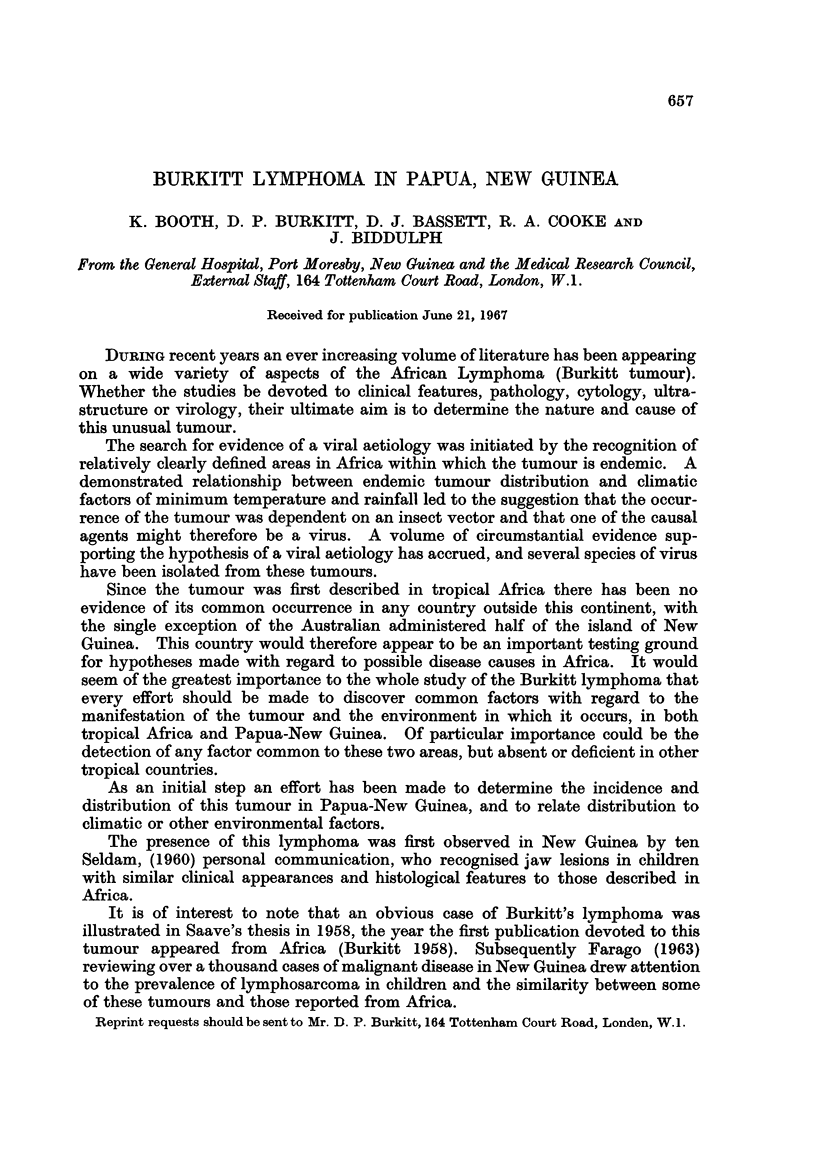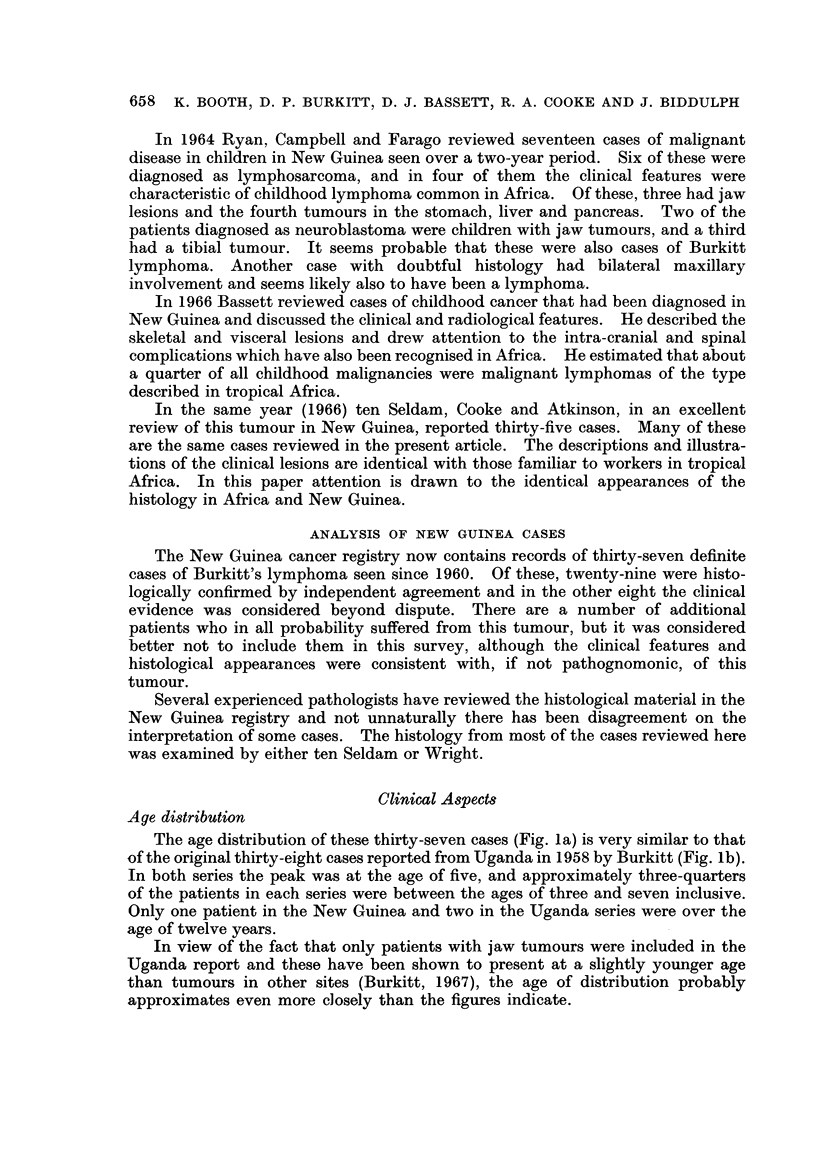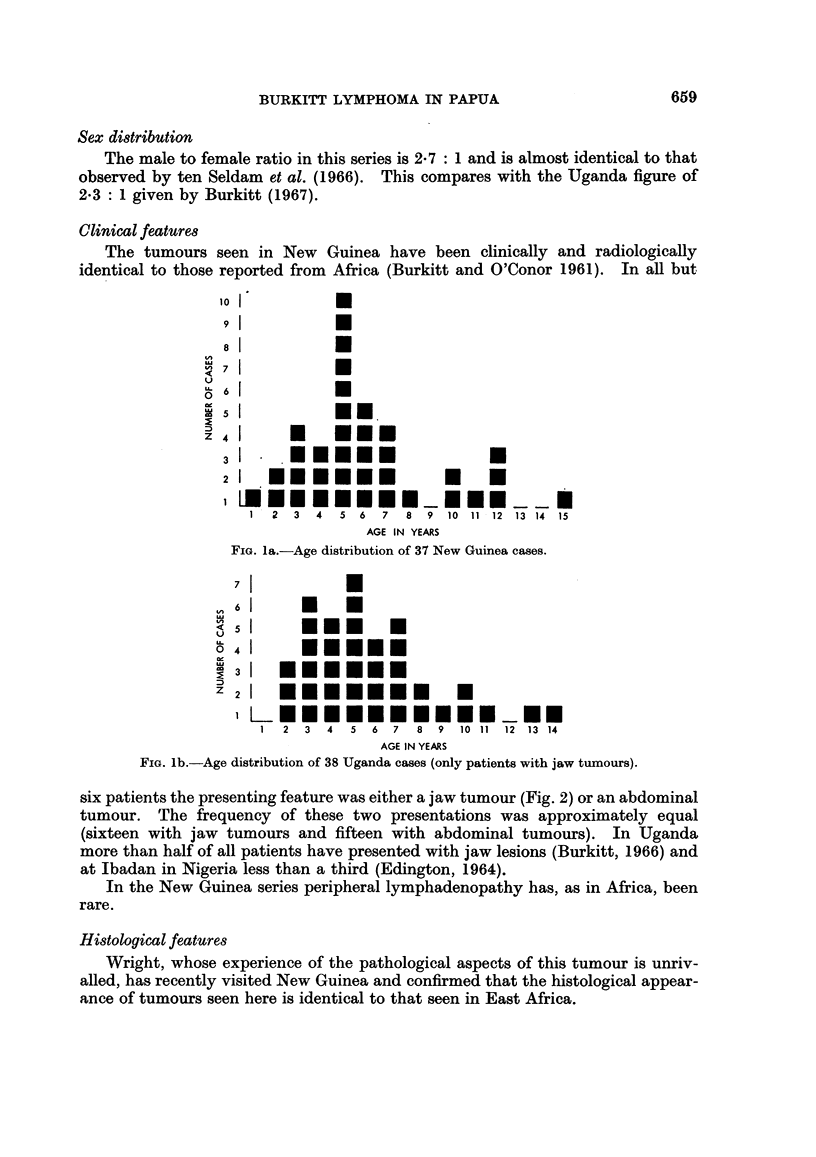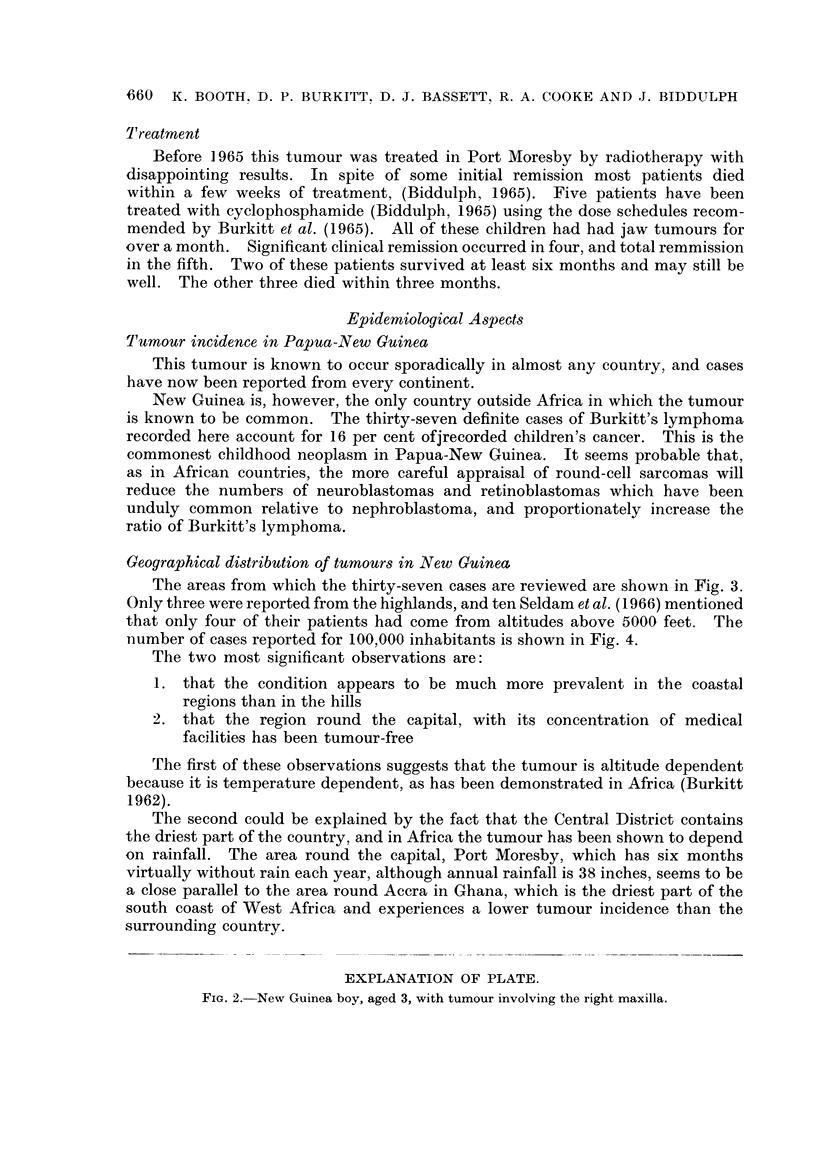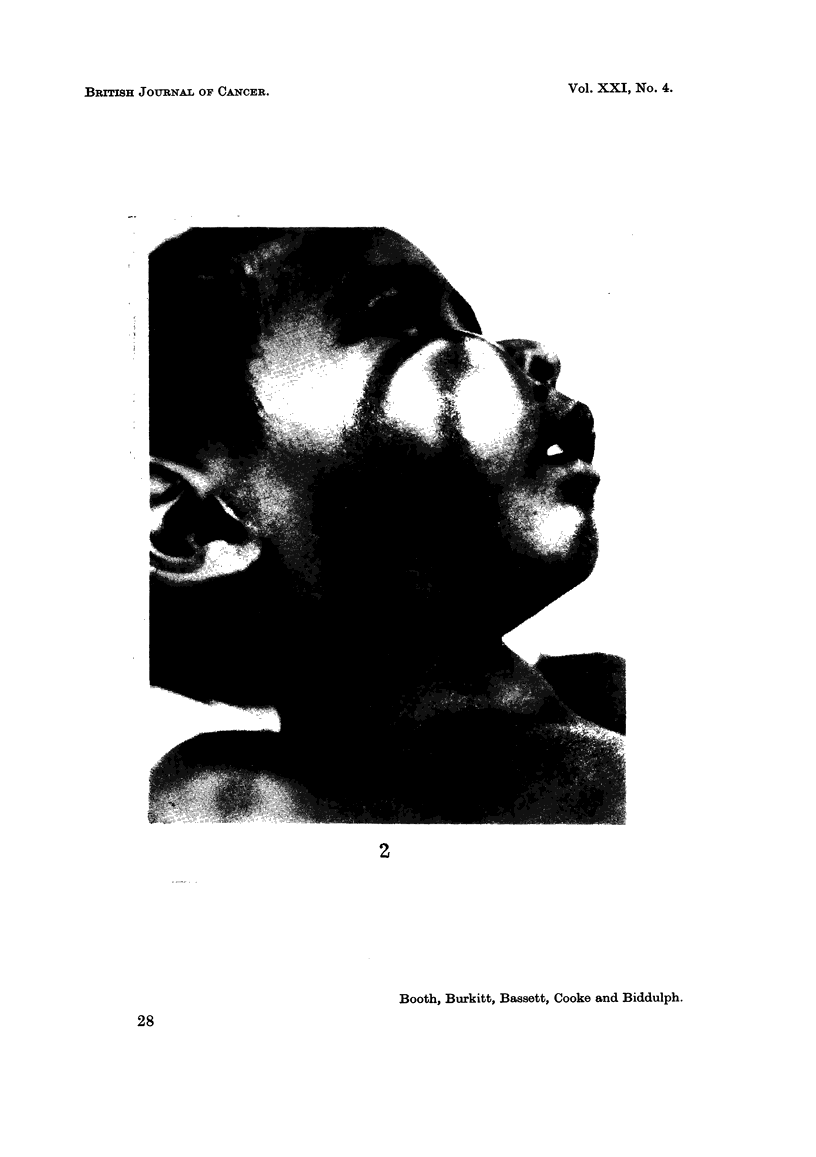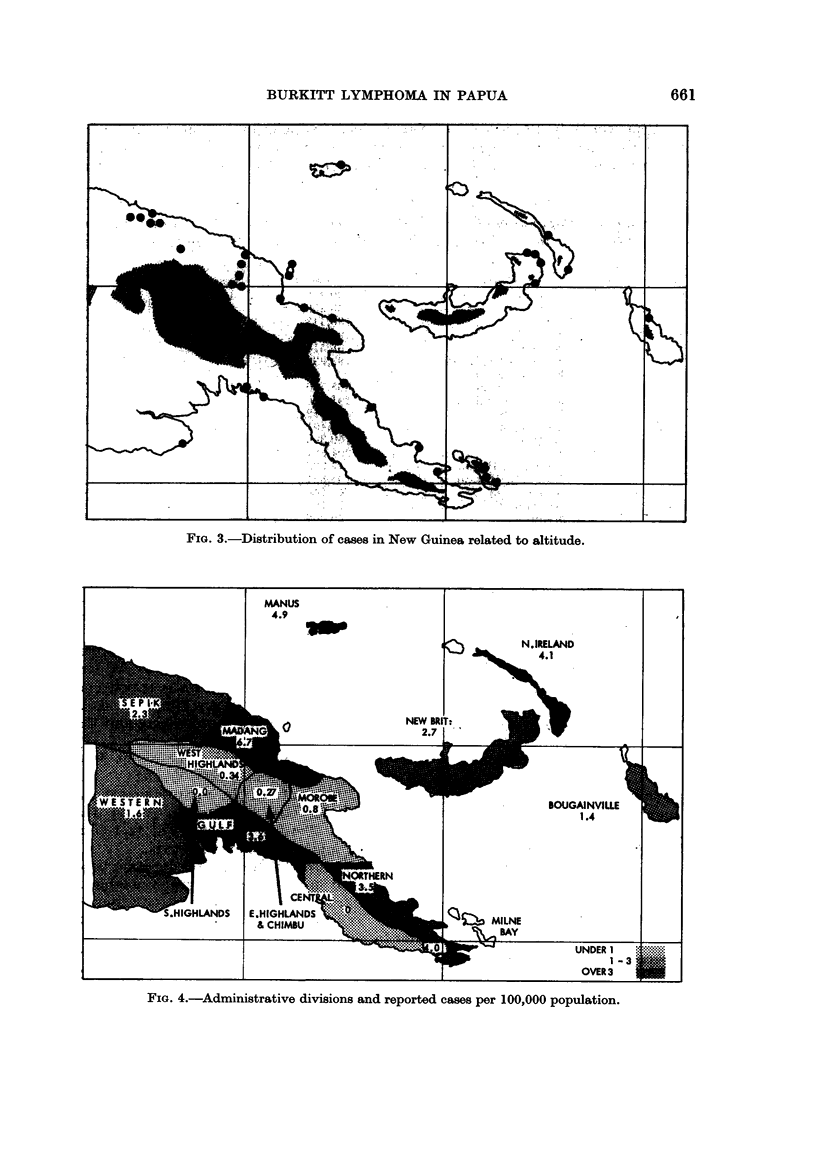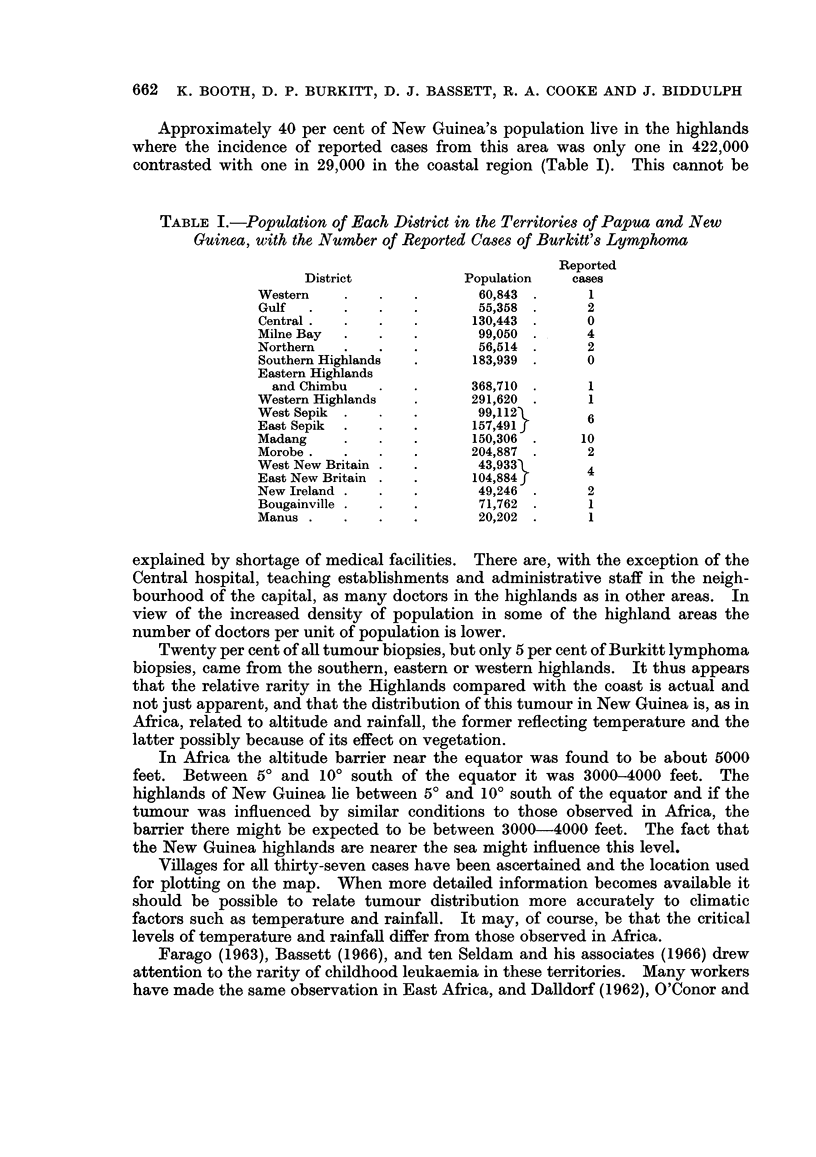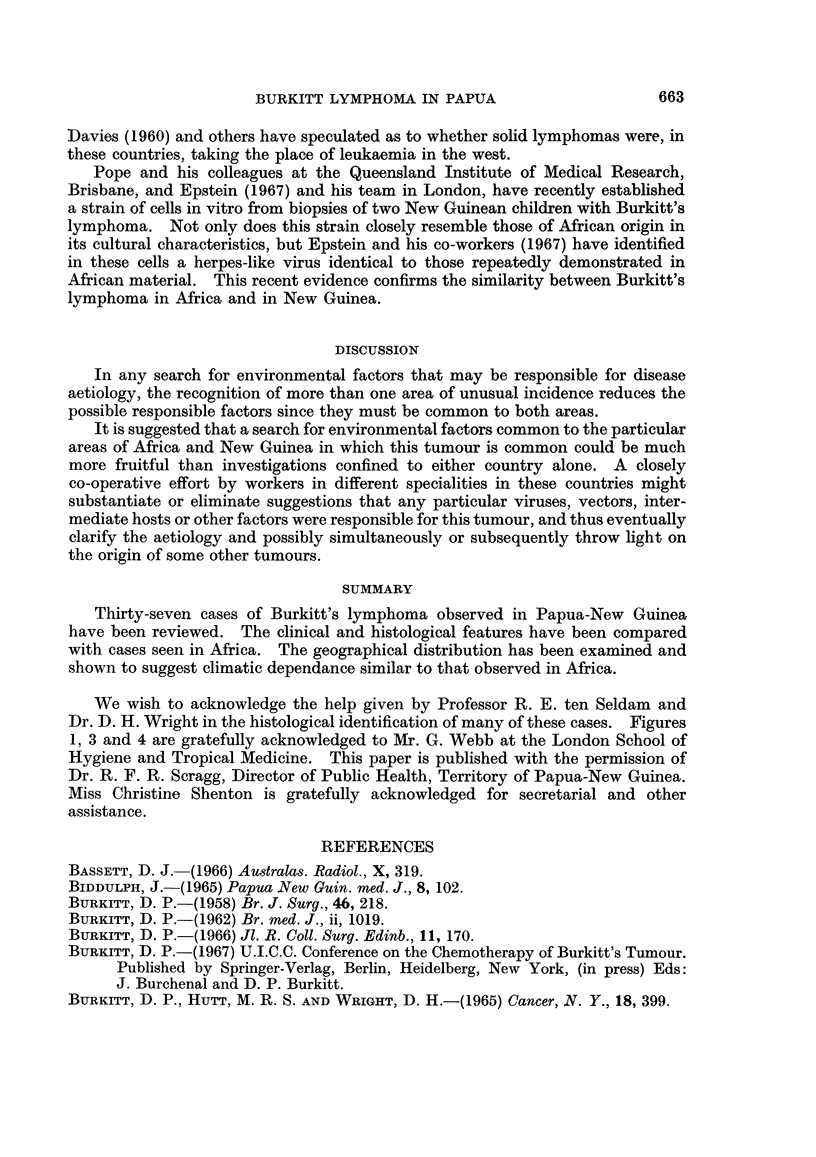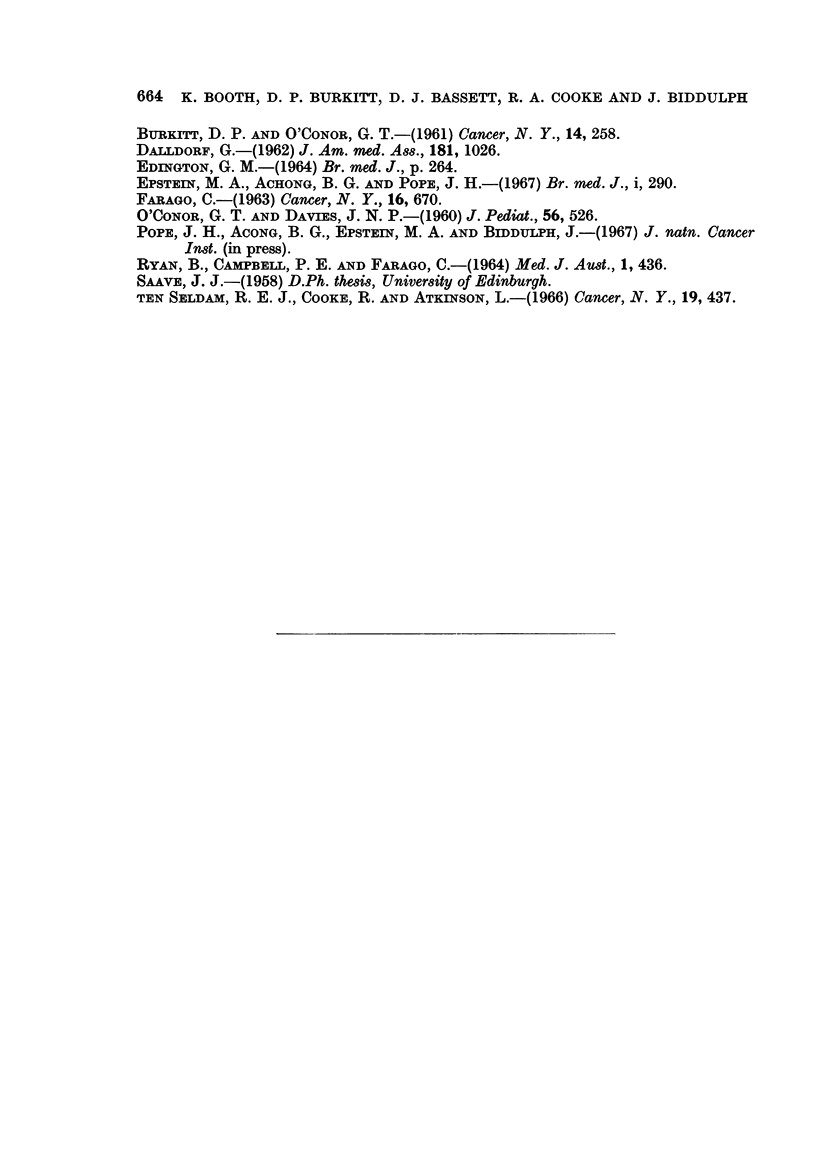# Burkitt lymphoma in Papua, New Guinea.

**DOI:** 10.1038/bjc.1967.77

**Published:** 1967-12

**Authors:** K. Booth, D. P. Burkitt, D. J. Bassett, R. A. Cooke, J. Biddulph

## Abstract

**Images:**


### BURKITT LYMPHOMA IN PAPUA, NEW GUINEA

K. BOOTH, D. P. BURKITT, D. J. BASSETT, R. A. COOKE AND J. BIDDULPH

*From the General Hospital, Port Moresby, New Guinea and the Medical Research Council, External Staff,* 164 *Tottenham Court Road, London, W.*1.

Received for publication June 21, 1967

DURING recent years an ever increasing volume of literature has been appearing on a wide variety of aspects of the African Lymphoma (Burkitt tumour). Whether the studies be devoted to clinical features, pathology, cytology, ultrastructure or virology, their ultimate aim is to determine the nature and cause of this unusual tumour.

The search for evidence of a viral aetiology was initiated by the recognition of relatively clearly defined areas in Africa within which the tumour is endemic. A demonstrated relationship between endemic tumour distribution and climatic factors of minimum temperature and rainfall led to the suggestion that the occurrence of the tumour was dependent on an insect vector and that one of the causal agents might therefore be a virus. A volume of circumstantial evidence supporting the hypothesis of a viral aetiology has accrued, and several species of virus have been isolated from these tumours.

Since the tumour was first described in tropical Africa there has been no evidence of its common occurrence in any country outside this continent, with the single exception of the Australian administered half of the island of New Guinea. This country would therefore appear to be an important testing ground for hypotheses made with regard to possible disease causes in Africa. It would seem of the greatest importance to the whole study of the Burkitt lymphoma that every effort should be made to discover common factors with regard to the manifestation of the tumour and the environment in which it occurs, in both tropical Africa and Papua-New Guinea. Of particular importance could be the detection of any factor common to these two areas, but absent or deficient in other tropical countries.

As an initial step an effort has been made to determine the incidence and distribution of this tumour in Papua-New Guinea, and to relate distribution to climatic or other environmental factors.

The presence of this lymphoma was first observed in New Guinea by ten Seldam, (1960) personal communication, who recognised jaw lesions in children with similar clinical appearances and histological features to those described in Africa.

It is of interest to note that an obvious case of Burkitt's lymphoma was illustrated in Saave's thesis in 1958, the year the first publication devoted to this tumour appeared from Africa (Burkitt 1958). Subsequently Farago (1963) reviewing over a thousand cases of malignant disease in New Guinea drew attention to the prevalence of lymphosarcoma in children and the similarity between some of these tumours and those reported from Africa.

Reprint requests should be sent to Mr. D. P. Burkitt, 164 Tottenham Court Road, Londen, W.1.

In 1964 Ryan, Campbell and Farago reviewed seventeen cases of malignant disease in children in New Guinea seen over a two-year period. Six of these were diagnosed as lymphosarcoma, and in four of them the clinical features were characteristic of childhood lymphoma common in Africa. Of these, three had jaw lesions and the fourth tumours in the stomach, liver and pancreas. Two of the patients diagnosed as neuroblastoma were children with jaw tumours, and a third had a tibial tumour. It seems probable that these were also cases of Burkitt lymphoma. Another case with doubtful histology had bilateral maxillary involvement and seems likely also to have been a lymphoma.

In 1966 Bassett reviewed cases of childhood cancer that had been diagnosed in New Guinea and discussed the clinical and radiological features. He described the skeletal and visceral lesions and drew attention to the intra-cranial and spinal complications which have also been recognised in Africa. He estimated that about a quarter of all childhood malignancies were malignant lymphomas of the type described in tropical Africa.

In the same year (1966) ten Seldam, Cooke and Atkinson, in an excellent review of this tumour in New Guinea, reported thirty-five cases. Many of these are the same cases reviewed in the present article. The descriptions and illustrations of the clinical lesions are identical with those familiar to workers in tropical Africa. In this paper attention is drawn to the identical appearances of the histology in Africa and New Guinea.

### ANALYSIS OF NEW GUINEA CASES

The New Guinea cancer registry now contains records of thirty-seven definite cases of Burkitt's lymphoma seen since 1960. Of these, twenty-nine were histologically confirmed by independent agreement and in the other eight the clinical evidence was considered beyond dispute. There are a number of additional patients who in all probability suffered from this tumour, but it was considered better not to include them in this survey, although the clinical features and histological appearances were consistent with, if not pathognomonic, of this tumour.

Several experienced pathologists have reviewed the histological material in the New Guinea registry and not unnaturally there has been disagreement on the interpretation of some cases. The histology from most of the cases reviewed here was examined by either ten Seldam or Wright.

### *Clinical Aspects*

#### *Age distribution*

The age distribution of these thirty-seven cases (Fig. 1a) is very similar to that of the original thirty-eight cases reported from Uganda in 1958 by Burkitt (Fig. 1b). In both series the peak was at the age of five, and approximately three-quarters of the patients in each series were between the ages of three and seven inclusive. Only one patient in the New Guinea and two in the Uganda series were over the age of twelve years.

In view of the fact that only patients with jaw tumours were included in the Uganda report and these have been shown to present at a slightly younger age than tumours in other sites (Burkitt, 1967), the age of distribution probably approximates even more closely than the figures indicate.

*Sex distribution*

The male to female ratio in this series is 2·7 : 1 and is almost identical to that observed by ten Seldam *et al.* (1966). This compares with the Uganda figure of 2·3 : 1 given by Burkitt (1967).

*Clinical features*

The tumours seen in New Guinea have been clinically and radiologically identical to those reported from Africa (Burkitt and O'Conor 1961). In all but

FIG. 1a.—Age distribution of 37 New Guinea cases.

FIG. 1b.—Age distribution of 38 Uganda cases (only patients with jaw tumours).

six patients the presenting feature was either a jaw tumour (Fig. 2) or an abdominal tumour. The frequency of these two presentations was approximately equal (sixteen with jaw tumours and fifteen with abdominal tumours). In Uganda more than half of all patients have presented with jaw lesions (Burkitt, 1966) and at Ibadan in Nigeria less than a third (Edington, 1964).

In the New Guinea series peripheral lymphadenopathy has, as in Africa, been rare.

*Histological features*

Wright, whose experience of the pathological aspects of this tumour is unrivalled, has recently visited New Guinea and confirmed that the histological appearance of tumours seen here is identical to that seen in East Africa.

*Treatment*

Before 1965 this tumour was treated in Port Moresby by radiotherapy with disappointing results. In spite of some initial remission most patients died within a few weeks of treatment, (Biddulph, 1965). Five patients have been treated with cyclophosphamide (Biddulph, 1965) using the dose schedules recommended by Burkitt *et al.* (1965). All of these children had had jaw tumours for over a month. Significant clinical remission occurred in four, and total remmission in the fifth. Two of these patients survived at least six months and may still be well. The other three died within three months.

### *Epidemiological Aspects*

*Tumour incidence in Papua-New Guinea*

This tumour is known to occur sporadically in almost any country, and cases have now been reported from every continent.

New Guinea is, however, the only country outside Africa in which the tumour is known to be common. The thirty-seven definite cases of Burkitt's lymphoma recorded here account for 16 per cent ofjrecorded children's cancer. This is the commonest childhood neoplasm in Papua-New Guinea. It seems probable that, as in African countries, the more careful appraisal of round-cell sarcomas will reduce the numbers of neuroblastomas and retinoblastomas which have been unduly common relative to nephroblastoma, and proportionately increase the ratio of Burkitt's lymphoma.

*Geographical distribution of tumours in New Guinea*

The areas from which the thirty-seven cases are reviewed are shown in Fig. 3. Only three were reported from the highlands, and ten Seldam *et al.* (1966) mentioned that only four of their patients had come from altitudes above 5000 feet. The number of cases reported for 100,000 inhabitants is shown in Fig. 4.

The two most significant observations are:

1. that the condition appears to be much more prevalent in the coastal regions than in the hills
2. that the region round the capital, with its concentration of medical facilities has been tumour-free

The first of these observations suggests that the tumour is altitude dependent because it is temperature dependent, as has been demonstrated in Africa (Burkitt 1962).

The second could be explained by the fact that the Central District contains the driest part of the country, and in Africa the tumour has been shown to depend on rainfall. The area round the capital, Port Moresby, which has six months virtually without rain each year, although annual rainfall is 38 inches, seems to be a close parallel to the area round Accra in Ghana, which is the driest part of the south coast of West Africa and experiences a lower tumour incidence than the surrounding country.

EXPLANATION OF PLATE.

FIG. 2.—New Guinea boy, aged 3, with tumour involving the right maxilla.

2

Booth, Burkitt, Bassett, Cooke and Biddulph.

**Fig. 3.—Distribution of cases in New Guinea related to altitude.**

**Fig. 4.—Administrative divisions and reported cases per 100,000 population.**

Approximately 40 per cent of New Guinea's population live in the highlands where the incidence of reported cases from this area was only one in 422,000 contrasted with one in 29,000 in the coastal region (Table I). This cannot be

TABLE I.—*Population of Each District in the Territories of Papua and New Guinea, with the Number of Reported Cases of Burkitt's Lymphoma*

| District | Population | Reported cases |
|---|---|---|
| Western | 60,843 | 1 |
| Gulf | 55,358 | 2 |
| Central | 130,443 | 0 |
| Milne Bay | 99,050 | 4 |
| Northern | 56,514 | 2 |
| Southern Highlands | 183,939 | 0 |
| Eastern Highlands and Chimbu | 368,710 | 1 |
| Western Highlands | 291,620 | 1 |
| West Sepik | 99,112 } | 6 |
| East Sepik | 157,491 } | |
| Madang | 150,306 | 10 |
| Morobe | 204,887 | 2 |
| West New Britain | 43,933 } | 4 |
| East New Britain | 104,884 } | |
| New Ireland | 49,246 | 2 |
| Bougainville | 71,762 | 1 |
| Manus | 20,202 | 1 |

explained by shortage of medical facilities. There are, with the exception of the Central hospital, teaching establishments and administrative staff in the neighbourhood of the capital, as many doctors in the highlands as in other areas. In view of the increased density of population in some of the highland areas the number of doctors per unit of population is lower.

Twenty per cent of all tumour biopsies, but only 5 per cent of Burkitt lymphoma biopsies, came from the southern, eastern or western highlands. It thus appears that the relative rarity in the Highlands compared with the coast is actual and not just apparent, and that the distribution of this tumour in New Guinea is, as in Africa, related to altitude and rainfall, the former reflecting temperature and the latter possibly because of its effect on vegetation.

In Africa the altitude barrier near the equator was found to be about 5000 feet. Between 5° and 10° south of the equator it was 3000–4000 feet. The highlands of New Guinea lie between 5° and 10° south of the equator and if the tumour was influenced by similar conditions to those observed in Africa, the barrier there might be expected to be between 3000—4000 feet. The fact that the New Guinea highlands are nearer the sea might influence this level.

Villages for all thirty-seven cases have been ascertained and the location used for plotting on the map. When more detailed information becomes available it should be possible to relate tumour distribution more accurately to climatic factors such as temperature and rainfall. It may, of course, be that the critical levels of temperature and rainfall differ from those observed in Africa.

Farago (1963), Bassett (1966), and ten Seldam and his associates (1966) drew attention to the rarity of childhood leukaemia in these territories. Many workers have made the same observation in East Africa, and Dalldorf (1962), O'Conor and

Davies (1960) and others have speculated as to whether solid lymphomas were, in these countries, taking the place of leukaemia in the west.

Pope and his colleagues at the Queensland Institute of Medical Research, Brisbane, and Epstein (1967) and his team in London, have recently established a strain of cells in vitro from biopsies of two New Guinean children with Burkitt's lymphoma. Not only does this strain closely resemble those of African origin in its cultural characteristics, but Epstein and his co-workers (1967) have identified in these cells a herpes-like virus identical to those repeatedly demonstrated in African material. This recent evidence confirms the similarity between Burkitt's lymphoma in Africa and in New Guinea.

### DISCUSSION

In any search for environmental factors that may be responsible for disease aetiology, the recognition of more than one area of unusual incidence reduces the possible responsible factors since they must be common to both areas.

It is suggested that a search for environmental factors common to the particular areas of Africa and New Guinea in which this tumour is common could be much more fruitful than investigations confined to either country alone. A closely co-operative effort by workers in different specialities in these countries might substantiate or eliminate suggestions that any particular viruses, vectors, intermediate hosts or other factors were responsible for this tumour, and thus eventually clarify the aetiology and possibly simultaneously or subsequently throw light on the origin of some other tumours.

### SUMMARY

Thirty-seven cases of Burkitt's lymphoma observed in Papua-New Guinea have been reviewed. The clinical and histological features have been compared with cases seen in Africa. The geographical distribution has been examined and shown to suggest climatic dependance similar to that observed in Africa.

We wish to acknowledge the help given by Professor R. E. ten Seldam and Dr. D. H. Wright in the histological identification of many of these cases. Figures 1, 3 and 4 are gratefully acknowledged to Mr. G. Webb at the London School of Hygiene and Tropical Medicine. This paper is published with the permission of Dr. R. F. R. Scragg, Director of Public Health, Territory of Papua-New Guinea. Miss Christine Shenton is gratefully acknowledged for secretarial and other assistance.

### REFERENCES

Bassett, D. J.—(1966) *Australas. Radiol.*, **X**, 319.
Biddulph, J.—(1965) *Papua New Guin. med. J.*, **8**, 102.
Burkitt, D. P.—(1958) *Br. J. Surg.*, **46**, 218.
Burkitt, D. P.—(1962) *Br. med. J.*, ii, 1019.
Burkitt, D. P.—(1966) *Jl. R. Coll. Surg. Edinb.*, **11**, 170.
Burkitt, D. P.—(1967) U.I.C.C. Conference on the Chemotherapy of Burkitt's Tumour. Published by Springer-Verlag, Berlin, Heidelberg, New York, (in press) Eds: J. Burchenal and D. P. Burkitt.
Burkitt, D. P., Hutt, M. R. S. and Wright, D. H.—(1965) *Cancer, N. Y.*, **18**, 399.

BURKITT, D. P. AND O'CONOR, G. T.—(1961) *Cancer, N. Y.*, **14**, 258.
DALLDORF, G.—(1962) *J. Am. med. Ass.*, **181**, 1026.
EDINGTON, G. M.—(1964) *Br. med. J.*, p. 264.
EPSTEIN, M. A., ACHONG, B. G. AND POPE, J. H.—(1967) *Br. med. J.*, i, 290.
FARAGO, C.—(1963) *Cancer, N. Y.*, **16**, 670.
O'CONOR, G. T. AND DAVIES, J. N. P.—(1960) *J. Pediat.*, **56**, 526.
POPE, J. H., ACONG, B. G., EPSTEIN, M. A. AND BIDDULPH, J.—(1967) *J. natn. Cancer Inst.* (in press).
RYAN, B., CAMPBELL, P. E. AND FARAGO, C.—(1964) *Med. J. Aust.*, **1**, 436.
SAAVE, J. J.—(1958) *D.Ph. thesis, University of Edinburgh.*
TEN SELDAM, R. E. J., COOKE, R. AND ATKINSON, L.—(1966) *Cancer, N. Y.*, **19**, 437.